# Taking care of patients with recessive dystrophic epidermolysis bullosa from birth to adulthood: a multidisciplinary Italian Delphi consensus

**DOI:** 10.1186/s13023-025-03635-1

**Published:** 2025-03-16

**Authors:** May El Hachem, Andrea Diociaiuti, Domenico Bonamonte, Michela Brena, Lucia Lospalluti, Cristina Magnoni, Iria Neri, Ketty Peris, Gianluca Tadini, Giovanna Zambruno, the Delphi Study Group, Francesca Bevilacqua, Tamara Caldaro, Domenico De Rose, Roberta Depenni, Alessandro Di Stefani, Antonella Diamanti, Raimondo Femino, Chiara Fiorentini, Andrea Gabusi, Angela Galeotti, Alessandra Gelmetti, Claudia Giavoli, Giuseppe Giudice, Sophie Guez, Nicola Laforgia, Mario Lando, Claudia Laterza, Laura Lucaccioni, Laura Massella, Giuseppe Palumbo, Chiara Parolo, Marisa Pugliese, Simone Reali, Simona Salera, Paolo Taurisano, Marco Tucci, Graziamaria Ubertini, Antonella Vimercati, Veronica Volante, Marco Zazza

**Affiliations:** 1 Dermatology Unit and Genodermatosis Unit, Translational Pediatrics and Clinical Genetics Research Area, Bambino Gesù Children’s Hospital-IRCCS, Piazza Sant’Onofrio, 4, 00165 Rome, Italy; 2 Department of Precision and Regenerative Medicine and Ionian Area, Unit of Dermatology, University of Bari “Aldo Moro”, Policlinico of Bari, Bari, Italy; 3 Pediatric Dermatology Unit, Department of Clinical Sciences and Community Health, Ca’ Granda Foundation - IRCCS, Ospedale Maggiore Policlinico, Milan, Italy; 4 Department of Surgical, Medical, Dental, and Morphological Sciences with a Focus on Transplantation, Oncology, and Regenerative Medicine, University of Modena and Reggio Emilia, Modena, Italy; 5 Dermatology Unit, University Hospital of Bologna-IRCCS, Bologna, Italy; 6 Dermatology Unit, Department of Translational Medicine and Surgery, Catholic University of the Sacred Heart, Rome, Italy; 7 Dermatology Unity, Department of Medical and Surgical Sciences, A. Gemelli University Hospital Foundation - IRCCS, Rome, Italy; 8 Center for Hereditary Skin Diseases, Pediatric Dermatology Unit, University of Milan, Ospedale Maggiore Policlinico, Milan, Italy

**Keywords:** Multidisciplinary management, Wound care, Squamous cell carcinoma, Gastrointestinal involvement, Nutrition, Hand and foot deformities, Osteoporosis, Renal involvement, Pain, Therapeutic education

## Abstract

**Background:**

Recessive dystrophic epidermolysis bullosa (RDEB) is a rare and severe mucocutaneous fragility disorder due to mutations in the *COL7A1* gene encoding collagen VII, the major constituent of anchoring fibrils essential for epithelial adhesion. RDEB is characterized by unremitting blistering, chronic painful wounds and fibrotic scarring that results in hand and foot pseudosyndactyly, microstomia, and esophageal strictures. RDEB complications include nutritional compromise, chronic anemia, failure to thrive, delayed puberty, osteoporosis, and renal involvement. In addition, early onset cutaneous squamous cell carcinomas (cSCC) represent the first cause of premature death. Despite recent progress in wound care, disease management still relies on symptomatic and preventive measures. No clinical practice guidelines specifically focused on the care of RDEB are currently available. The present multidisciplinary consensus recommendations were generated following a modified Delphi method with the aim to provide healthcare professionals with practical statements on RDEB management from birth to adulthood.

**Results:**

Ten experts from six Italian EB reference centers developed 86 statements based on existing clinical practice guidelines and consensus recommendations for EB, literature data, and personal expertise. A multidisciplinary group of 30 members, representative of all major specialties relevant to RDEB management, participated to the anonymous online voting process. All statements reached consensus (> 75% agreement) at first voting round. Statements are divided into four major areas: (1) diagnosis, (2) neonatal age and infancy, (3) from childhood to adulthood, and (4) transversal age-independent issues, each of the last three comprising multiple domains of care. In particular, the section on patient care from childhood to adults deals with measures for management of wounds, gastrointestinal, eye and renal involvement, nutritional compromise, anemia, hand and foot deformities, cSCC, delayed puberty and osteoporosis, sexuality, pregnancy and delivery. Transversal issues comprise: pain and itch management, patient care in the operating theatre, physiotherapy and occupational therapy, therapeutic patient education and psychosocial support.

**Conclusions:**

The proposed practical and synthetic recommendations cover all major issues in the management of patients with RDEB from birth to adulthood. They can represent a useful tool to support hospital healthcare personnel as well as primary care physicians in the complex multidisciplinary management of RDEB.

**Supplementary Information:**

The online version contains supplementary material available at 10.1186/s13023-025-03635-1.

## Background

Inherited epidermolysis bullosa (EB) refers to a clinically and genetically heterogeneous group of rare disorders characterized by fragility and blistering of the skin and mucous membranes. Four main forms of EB are identified based on the level of blister formation within the skin: EB simplex (EBS), junctional EB (JEB), dystrophic EB (DEB), and Kindler EB (KEB) [1]. EBS, JEB and DEB are further classified into subtypes according to the inheritance mode and a mixture of immunofluorescence, ultrastructural, molecular, and phenotypic traits [2]. EB phenotypes are extremely diverse, ranging from localized forms that present primarily acral skin involvement to severe or syndromic subtypes. In the latter, complications due to generalized skin and mucosal fragility (e.g. sepsis, failure to thrive) or extracutaneous organ involvement (e.g. cardiomyopathy, muscular dystrophy, pyloric atresia) can result in reduced life expectancy [1, 3].

DEB is the second commonest type of EB, after EBS [4–6]. All DEB subtypes are caused by alterations in the *COL7A1* gene encoding collagen VII, the primary constituent of the anchoring fibrils that ensure adhesion of stratified epithelia to the underlying mesenchyme. DEB can be inherited as a recessive or dominant trait; phenotypic overlap between the two subtypes is significant, but in general, recessive dystrophic EB (RDEB) is more severe than the dominant disease. Clinically, DEB is characterized by blistering followed by scarring in the skin and mucosae of the gastrointestinal and genito-urinary tracts, and the eye. In particular, RDEB is hallmarked by unremitting blistering and defective wound healing, leading to chronic painful and easily infected wounds with inflammation and progressive fibrosis [7, 8]. Fibrotic processes result in pseudosyndactyly and mitten deformities of hands and feet and joint contractures, but also underlie a number of extracutaneous complications, such as microstomia, esophageal and anal strictures, which in turn contribute to nutritional compromise, chronic anemia and failure to thrive [3, 9]. In addition, fibrosis is one of the factors that may contribute to the development of highly aggressive cutaneous squamous cell carcinomas (cSCCs), which represent the leading cause of premature death in RDEB [3, 9, 10]. Overall, patients with RDEB experience major impairment of their quality of life (QoL) due to disease progression and complications, functional and esthetic damages, and great discomfort [9]. RDEB humanistic burden also heavily involves patient families and caregivers. Finally, RDEB care implies high direct and indirect economic costs for patients and their families [9]. The severity and burden of RDEB have fostered the development of different approaches of molecular and cell-based therapy as well as disease-modifying and symptomatic control agents, some of which have entered or even completed clinical trials [11]. Specific to wound care, a gel containing a birch bark extract has been recently authorized in Europe and the U.S. for topical treatment of wounds in individuals affected with JEB and DEB from 6 months of age [12]. In addition, the U.S. Food and Drug Administration (FDA) has approved the first topical gene therapy–based gel for the treatment of DEB wounds [13]. Despite remarkable progress, there is still no cure for RDEB and disease management relies on symptomatic and preventive measures, as well as early detection and treatment of complications [11].

Consensus recommendations and best practice guidelines have been published on different aspects of EB care or patient ages, but none has been specifically centered on RDEB [14–28]. A group of experts from Italian EB reference centers, which are also members of the European Reference Network for Rare and Undiagnosed Skin disorders (ERN-Skin, https://ern-skin.eu/), developed the present multidisciplinary practical consensus recommendations focused on the management of patients with RDEB from birth to adulthood. The aim of these recommendations is to support clinicians in RDEB care and to improve equity in healthcare and QoL of these difficult-to-treat and complex patients and their families.

## Methods

### Design

For this study, a modified Delphi consensus methodology was used. This procedure is a well-established instrument for reaching consensus between a panel of experts for research questions that cannot be answered with empirical evidence and complete certainty [29, 30]. Following the development of structured statements by a group of experts based on their expertise and a focused literature review, an iterative technique starts based on the scoring of the statements by a larger expert panel. The process is repeated in multiple rounds until consensus has been reached [31]. This method has been widely employed in the rare disease field due to the low level of evidence of most available studies [32, 33].

### Participants

Ten experts from six Italian EB reference centers developed and wrote the statements. Five out of six reference centers are also members of the ERN-Skin. The Delphi Study Group participating to the voting process comprised 30 members, representative of all major specialties involved in the management of individuals with RDEB: 2 neonatologists, 2 pediatricians, 3 dermatologists, 2 nutritionists/dietitians, 3 anesthesiologists/pain therapists, 1 digestive surgeon, 1 plastic surgeon, 2 hand surgeons, 2 endocrinologists, 1 ophthalmologist, 1 hematologist, 2 dentists, 1 gynecologist, 1 nephrologist, 2 oncologists, 1 physiotherapist, 3 psychologists.

### Elaboration of recommendation statements

Four major areas were identified: (1) Diagnosis, (2) Neonatal age (up to 28 days past expected due date) and infancy (from 28 days to one year), (3) From childhood (from 1 year to puberty) to adulthood (from 18 years onwards), and (4) Transversal age-independent issues—each of the last three comprising multiple domains of care. During the period November 2022–November 2023, experts generated statements based primarily on existing clinical practice guidelines and consensus recommendations, and, for topics not dealt with in available guidelines/recommendations, on literature review of articles published from 2005 till December 2022 (a search update was performed in July 2023), as well as their expertise in the management and treatment of RDEB. Consensus statements were discussed by expert panel members during a half-day virtual meeting, modified, and forwarded by e-mail to all panel members for approval. Final statements were shared with the Delphi Study Group for voting.

### Data collection

An online Delphi procedure was performed. All statements were voted anonymously by Delphi Study Group members using a dedicated online platform available for one month (from December 2023 to January 2024). Voting members were e-mailed a unique link to the voting platform. The results of the voting per each statement were analyzed by the expert panel members at the end of the round.

### Voting process

Eighty-six statements were voted by Delphi Study Group members. All members participated to the first voting phase, expressing their degree of agreement on a 5-point Likert scale (1 = strongly disagree; 2 = disagree; 3 = partially agree; 4 = agree; 5 = strongly agree). The definition of consensus was set at ≥ 75% agreement for 4 + 5 scores [34], and it was met at the first round.

## Results

All the proposed statements reached consensus, defined as ≥ 75% agreement for scores 4 + 5. A total of 61 statements reached 100% agreement, 16 statements 97% agreement, 1 statement 94%, 6 statements 93% and 2 statements 90% [see Additional file 1, Table S1].

Consensus statements for each area are presented below, together with a brief explanation based on literature findings and expert opinions. Details on agreement percentages for individual statements are listed in Additional file 1, Table S1.

**1. General premise**

RDEB is characterized by mucocutaneous blistering that appears at birth or in the first days of life and requires specific and adapted care measures, starting from the neonatal period. To carry out appropriate diagnosis and treatment and promptly start therapeutic parent education, a coordinated multidisciplinary management must be adopted as early as possible, involving trained personnel from specialized reference centers [15]. Multidisciplinary care should be continued lifelong.

*STATEMENT 1—Care of RDEB patients, from diagnosis to treatment, should be performed in reference centers offering coordinated multidisciplinary management by trained personnel.*

**2. Diagnosis**

When a neonate or infant develops blisters and erosions, the diagnosis of EB must be always considered, and diagnostic work-up promptly performed, following history taking and complete physical examination. An early diagnosis allows to plan adequate care, inform parents about prognosis and start therapeutic education [2].

Similar to all other EB types, the diagnosis of RDEB is based on a combination of clinical features, family history and laboratory work-up that includes a skin biopsy for immunofluorescence antigen mapping (IFM), and blood sampling for molecular genetic testing [2, 35]. IFM allows definition of the cleavage plane and thus diagnosis of the major EB type. It also permits assessment of the absence or reduced/altered expression of different proteins analysed, with implications for the diagnosis of specific EB subtypes. Of note, IFM results can be delivered in very short time. Transmission electron microscopy examination of a skin biopsy may be relevant to the diagnosis of specific DEB subtypes and should also be performed, whenever the technique and expertise are available. Molecular genetic testing represents the gold standard for EB diagnosis: it allows genetic counselling and DNA-based prenatal diagnosis [2, 35].

The dermatologist, neonatologist or paediatrician, medical geneticist, and psychologist should be involved in the diagnosis communication process. The information should be delivered gradually to both parents and adapted to socio-cultural level of the family [15].

*STATEMENT 2—Diagnosis must be performed as early as possible in order to initiate the most appropriate treatment.*

*STATEMENT 3—The diagnosis of RDEB is based on a combination of clinical features, family history and laboratory findings, including immunofluorescence antigen mapping on a skin biopsy and molecular genetic testing.*

*STATEMENT 4—Genetic testing enables genetic counselling and DNA-based prenatal diagnosis.*

*STATEMENT 5—The communication of the diagnosis should involve the dermatologist, the neonatologist/pediatrician and, for genetic diagnosis, the medical geneticist. It should be addressed to both parents, adapting the information to the family socio-cultural level. A psychologist should support the family.*

**3. Neonatal age and infancy**

**3.1 Management of RDEB newborns and infants: general measures**

Routine procedures performed in RDEB newborns and infants can severely injury their fragile mucocutaneous tissues and cause lesions, which in turn contribute to severe complications, such as recurrent infections, dehydration and electrolyte imbalance, feeding difficulty, and failure to thrive [15, 34]. For these reasons, management of RDEB newborns and infants requires specific and adapted measures by trained personnel. A neonatologist/pediatrician, a dermatologist, an anesthesiologist, an ophthalmologist, a dentist, a psychologist, and specialized nurses are essential members of the multidisciplinary team [15]. Parents are an integral part of the team: they should be gradually and regularly trained in the care of their child from the first days of life (see paragraph 5.4) [15, 36, 37].

The following measures should be adopted to limit blistering [15, 36, 38]**:**

- place identification band over a protective dressing or use a foam one,
- secure the umbilical cord with a ligature,
- limit the use of incubators to rare conditions such as preterm neonates or transient need for oxygen administration, since humidity and heat can favor blistering,
- provide affected newborns with cushioning in their bed, and decrease pressure on the skin,
- avoid friction to prevent skin damage from shearing forces, by lifting rather than sliding the baby during handling,
- avoid the use of adhesive and sticky materials, as their removal can cause new lesions. In case of accidental application, silicone medical adhesive removers (SMAR) (e.g. Niltac®) should be employed for atraumatic removal.
- use soft silicone fixation tapes providing atraumatic removal (e.g. Mepitac®) to secure devices such as electrodes, catheters, tubes, and probes,
- use clip sensors covered with non-adherent silicone dressings to record heart rate by pulse oximetry,
- use thick padding before applying the blood pressure cuff,
- avoid naso- and oro-pharyngeal suction. If required, a small, soft and lubricated tube should be used, and minimal suction pressure exerted.

The following procedures are recommended in severely affected RDEB newborns/infants [15]:

- adequate analgesia should be administered regularly, and supplemented before each procedure,
- blood sampling for complete blood count, electrolytes, C-reactive protein, urea, creatinine, total serum protein and albumin, iron, zinc, and, whenever required, blood cultures,
- swabs for culture from suspicious/infected wounds,
- a venous access should be guaranteed.

Vaccines should be regularly administered according to the immunization schedule recommended in infancy.

*STATEMENT 6—Members of the multidisciplinary team usually involved in RDEB care in infancy are neonatologist/pediatrician, dermatologist, nutritionist/dietitian, anesthesiologist, ophthalmologist, dentist, psychologist and specialized nurses. Parents should be gradually and regularly trained in the care of their child.*

*STATEMENT 7—In RDEB newborns/infants, all cautions should be taken to reduce friction risk and minimize neonate/infants handling (lift the baby on a mattress and avoid sliding, choose appropriate garments, *etc*.); specific measures should be applied for routine interventional procedures (e.g. limit incubator use, avoid adhesive tapes, secure small electrodes with non-adhesive dressings, thick padding below the blood pressure cuff, whenever possible avoid naso- and oro-pharyngeal suction).*

*STATEMENT 8—In severely affected RDEB newborns, (1) blood sampling for hematology and biochemistry tests, and (2) swabs of suspicious/infected wounds for culture should be performed, and (3) a venous access should be guaranteed.*

**3.2 Management of RDEB newborns and infants: skin care**

Garment choice should also conform to the principle of limiting friction and rubbing. Front-fastening babygros can be used and underwear should be turned inside-out to avoid seam skin rubbing. The elastic edges of disposable nappies should be trimmed-off; alternatively, nappies should be lined with a soft material (e.g. soft silicone contact layer or foam such as Mepitac®, Mölnlycke). The diaper region should be cleaned using an oil- or emollient-based cleanser, or with white soft and liquid paraffin in equal part [15, 38].

It is advised to bath the baby in tepid to slightly warm water; the frequency will vary depending on the cutaneous and general conditions. If bathing is not possible, the baby or infant should be padded with saline solution or an oil-based cleanser [15].

For blister and wound care, cautions and recommendations are similar to those applying to older RDEB patients (see paragraph 4.2), including the use of the topical gel containing birch triterpenes recently approved to treat wounds in RDEB patients from 6 months of age [12]. In addition to frequent evaluation of response to dressings, the following specific measures should be implemented:

- particular attention should be paid to hand and foot wound dressing from the first days of life to delay digit fusions [18],
- for the diaper area, less exuding erosions can be treated with paraffin-impregnated gauzes, replaced at each nappy change, whereas silicone foams (e.g. Mepilex® or Mepilex®Transfer) are indicated in more exuding lesions.

Adequate analgesia must be administered before bathing and wound care (see paragraph 5.1).

*STATEMENT 9—Adequate analgesia must be administered before bathing and wound care.*

*STATEMENT 10—The diaper area requires specific protective (lining of disposable nappies with soft material) and cleansing measures (e.g. with liquid and white soft paraffin in equal parts or with an emollient/oil-based cleanser). Wounds should be managed according to principles applied for other body areas; however, paraffin-impregnated gauzes represent a cost-effective alternative.*

*STATEMENT 11—Particular attention should be paid to hand and foot wound dressing from the first days of life to delay digit fusions.*

**3.3 Management of RDEB newborns and infants: feeding, discharge and follow-up**

In addition to their normal dietary demands, newborns and infants with severe RDEB have increased nutritional requirements related to fluid and protein losses through wounds [36, 39].

For neonates and infants with mild to moderate oral involvement, breast-feeding is recommended [15, 39]:

- the mother should be encouraged and trained to breast-feed her baby,
- soft paraffin can be applied on the nipple and breast as well as on the infant face and lips to reduce friction from rooting reflex.

When breast-feeding is not possible (e.g. severe oral fragility, insufficient amount or flow of milk), oral feeding remains the best option, also allowing to add supplements in malnourished infants [39]. Commercially available teats should be softened with warm boiled water. The teat hole can be enlarged, or extra holes may be created to facilitate sucking. Lips can be protected with petroleum jelly to avoid the skin sticking to the teat.

Measures to prevent/treat constipation should be started early in infancy. They include extra-fluid (water or diluted fruit juice) administration, fiber-containing proprietary feed, and stool softener if needed.

Whenever possible nasogastric feeding should be avoided, as nasogastric tubes may result in further oro-pharyngeal and esophageal lesions [36]. If required, nasogastric feeding can be employed short-term using a small soft lubricated polyurethane tube to minimize internal trauma and reduce damage [15, 39].

Gastrostomy should be considered in babies who exhibit poor growth, oral health issues, severe persistent constipation, or elevated stress levels during feeding in order to improve the nutritional and health status as well as the QoL of patients and caregivers [40]. Although, gastrostomy-related complications are frequent, in particular around the stoma (e.g. excessive leakage, pain, erosion, chronic wound, and granulation tissue), they lead to G-tube removal in a few cases.

The infant should be discharged home when the general health condition is stable, and the parents are adequately trained and confident in caring for their baby (see paragraph 5.4). The emotional and financial effects of EB should also be taken into account for a successful transfer home, which may require the support of a social worker, psychologist, and specialized nurse [36]. Continuity of care from hospital discharge to community care is essential for successful management of RDEB patients.

A first follow-up visit with the specialized team (usually: dermatologist, pediatrician, EB nurse, psychologist) will be organized in 2–4 weeks. It should comprise a complete clinical examination (skin and mucosae, growth, nutrition, pain, etc.), dressing, and evaluation and pursue of the therapeutic education of the parents. If the infant care is correctly performed, the next appointment should be in one month, then every three months during infancy. In mild RDEB subtypes, follow-up visits can be every 6 months [15].

*STATEMENT 12—Breastfeeding is encouraged: appropriate measures should be taken to reduce friction from rooting reflex (lubricate nipple and breast as well as infant lips and face). For oral feeding, commercially available teats should be adapted (enlarged or extra holes, softening with warm water) to facilitate sucking.*

*STATEMENT 13—Whenever possible nasogastric feeding should be avoided. If required, it can be employed only in the short-term using a small soft lubricated polyurethane tube. In severe cases, gastrostomy should be considered to ensure adequate nutritional status since infancy.*

*STATEMENT 14—The infant should be discharged from the hospital when the general health condition is stable, and the parents are adequately trained and confident in caring for their baby.*

*STATEMENT 15—A first follow-up visit with the specialized team (usually: dermatologist, pediatrician, EB nurse, psychologist) should be organized in 2–4 weeks. Next appointment will usually be after one month, then every three months during infancy.*

**4. From childhood to adulthood**

**4.1 Follow-up: general measures and timing**

Regular follow-up is required lifelong to evaluate mucocutaneous involvement, disease complications, and general health status. Follow-up planning should also take into account family and patient compliance, specific needs and problems [37].

RDEB patients should be seen at least twice a year. More frequent follow-up visits (e.g. every 2–3 months) are required for the most severely affected patients or those with poor compliance in order to monitor and treat disease complications (e.g. chronic wounds, dental care, malnutrition, anemia, dysphagia, musculoskeletal deformities, osteoporosis, pain/itching) as well as to early diagnose and treat cSCCs [15].

The multidisciplinary team for the follow-up of children and adults comprises the dermatologist, pediatrician/internist, dentist, nutritionist, dietitian, specialized nurse, psychologist, and physiotherapist [37]. Additional specialists who may be involved on a case-by-case basis are the ophthalmologist, pain therapist, anesthesiologist, gastroenterologist, interventional radiologist/pediatric/digestive surgeon, oncologist, endocrinologist, cardiologist, nephrologist, orthopedist, plastic surgeon, ENT specialist, and speech therapist. Psychological problems and social relationships should be regularly evaluated with the support of the psychologist and social worker. Caregiver and patient compliance and their skills in wound care should be always taken into account in designing the care plan, and adherence to treatment regularly checked (see paragraph 5.4) [15, 37].

Monitoring of RDEB patients comprises at least once a year blood testing including a complete blood count, electrolytes, total serum protein and albumin, iron, iron-binding capacity, ferritin, erythrocyte sedimentation rate, C-reactive protein, liver function tests, urea, creatinine and, if required, zinc, selenium, folate and vitamins. In addition, immunization schedule for infectious diseases should be regularly continued [15].

When RDEB patients reach adulthood, particular attention should be given at the transition from the pediatric to the adult reference center, in order to warrant the continuity of care and preserve patients’ QoL as much as possible.

*STATEMENT 16—After infancy, follow-up should be scheduled at least every 6 months. It can be more frequent depending on specific needs and disease complications.*

*STATEMENT 17—Each follow-up visit should include evaluation of skin and mucosal involvement, general health status, adherence to treatment, and disease complications (e.g. chronic wounds, chronic pain/itching, dysphagia, anemia/malnutrition) and should be performed in the context of a multidisciplinary team including a dermatologist, pediatrician/internist, dentist, nutritionist, dietitian, specialized nurse, and physiotherapist. Additional specialists who may be involved on a case-by-case basis are ophthalmologist, pain therapist, anesthesiologist, interventional radiologist, pediatric digestive surgeon, gastroenterologist, oncologist, endocrinologist, cardiologist, nephrologist, orthopedist, plastic surgeon, Ear, Nose and Throat (ENT) specialist, and speech therapist. Psychological problems and social relationships should also be evaluated with the support of the psychologist and social worker*.

*STATEMENT 18—Immunization schedule for infectious diseases should be regularly continued*.

*STATEMENT 19—Particular attention should be given at the transition from the pediatric to the adult reference center, in order to warrant the continuity of care and possibly preserve patients’ quality of life.*

**4.2 Wound care**

Preventive measures, wound care and early detection and treatment of complications are the cornerstones of RDEB patient management. Preventive measures to reduce blister formation include: wearing proper footwear and hosiery (such as silver-lined socks), padding trauma-exposed areas, avoiding tight clothing or clothes with raised seams or labels [20, 23]. To avoid blister peripheral extension, new blisters should be lanced with a sterile needle (or finger prick lancet), leaving the blister roof in place [14, 38].

Appropriate wound management should consider [14, 23]:

- disease severity and patient age,
- nutritional status, chronic anaemia and hypoalbuminaemia,
- wound characteristics including inflammation and infection,
- associated symptoms, specifically pain and itch,
- impact on patient’s everyday life,
- adherence to treatment**.**

A personalized wound care plan should also take into account patient/caregiver preferences, psychological factors, and cost-effectiveness. Treatment compliance should be routinely monitored [14, 23].

Appropriate analgesia must be administered before dressing changes (see paragraph 5.1). All products and materials should be prepared and close at hand before initiating dressing [15, 23]. Wounds should be cleaned with sterile saline or low-toxicity antiseptic solutions (e.g. polyhexanide, chlorhexidine). Petrolatum should be applied repeatedly to gently eliminate crusts, then the area should be soaked and bathed [23]. The choice of dressings should consider the wound characteristics (site and size, exudate, critical colonization/infection), patient age, and patient/parent preferences [23].

In the absence of infection, wound dressing should be performed 2–3 times per week, preferably using advanced non-adherent dressings, such as soft silicone foams and polymeric membranes for exuding wounds, and soft silicone or lipido-colloid contact layers and hydrogels for dry/slightly exuding wounds [23]. When advanced dressings are not available, paraffin-impregnated gauzes can be used and should be changed every day. A soft roll gauze should be placed over the contact layer. Finally, tubular meshes can be used to secure underlying dressings [36]. Individual digit dressing initiated in infancy should be continued to delay digit fusion. The separation should be performed by using easily modelled dressings, such as contact layers or soft silicone foams [23].

The recently approved topical gel containing birch triterpenes should be applied directly on wounds or on primary dressings to accelerate wound healing in patients aged six months and older [12]. If available, an additional therapeutic option for treatment of RDEB wounds is the topical gene therapy gel, carrying the COL7A1 gene [13].

Clinical signs and symptoms of wound infection comprise: increasing size, exudate, odour, and pain, surrounding erythema and swelling [23]. Treatment of critically colonized and infected wounds requires specific measures [23]:

- skin swabs should be taken for microorganism identification and antimicrobial sensitivity determination. In case of multiple infected wounds and/or associated systemic symptoms, blood cultures should be obtained to rule out sepsis,
- wound dressing should be performed daily preferably using silicone-based foam dressings as contact layers,
- cleansing with mild antiseptics and application of an antiseptic cream (such as lipid-stabilized hydrogen peroxide) can reduce bacterial load,
- topical antibiotics/antimicrobials (e.g. fusidic acid, mupirocin) should be used for short periods to prevent resistance and sensitization. Alternatively, silver-containing products can be employed, paying attention to limit the time of administration and treated surface due to potential systemic absorption,
- systemic antibiotics should be administered in the presence of multiple infected lesions and/or deep infection with surrounding tissue involvement.

Healing of infected wounds should be closely monitored by clinical evaluation of wound (exudate) and perilesional area (erythema and swelling) appearance, odour, improvement of local symptoms (pain), and then size reduction [14]. In view of the known difficulty in full bacterial eradication in RDEB wounds, repeated swabs are mainly indicated in case of clinically non-responsive infection [23].

*STATEMENT 20—Wound care is the cornerstone of RDEB patient treatment. The wound care plan should be individually tailored and consider psychosocial aspects and patient preferences as well as cost effectiveness. Patient and caregiver should be trained, and adherence to treatment should be regularly checked.*

*STATEMENT 21—Appropriate analgesia must be administered before dressing changes, which should be performed in a relaxing environment with all dressing materials ready for use.*

*STATEMENT 22—The choice of dressings should consider the wound characteristics (site, and size, exudate, critical colonization/infection), patient age, and patient/parent preference.*

*Statement 23—In the absence of infection, wound dressing should be performed 2–3 times per week, using advanced non-adherent primary dressings, such as soft silicone foams and polymeric membrane dressings for exuding wounds, and soft silicone or lipido-colloid contact layers and hydrogels for dry/slightly exuding wounds. When advanced dressings are not available, paraffin-impregnated gauzes can be used and should be changed daily.*

*STATEMENT 24—Topical treatments comprise: (1) liquid paraffin for gentle crust removal, and (2) birch triterpenes gel for wounds.*

*STATEMENT 25—Individual digit dressing initiated in infancy should be continued to delay digit fusion. The separation should be performed by using easily modelled dressings, such as contact layers or soft silicone foams.*

*STATEMENT 26—Treatment of critically colonized and infected wounds requires specific measures: (1) skin swab should be taken for microorganism identification and antimicrobial sensitivity determination, (2) wound dressing should be performed daily using silicone-based foam dressings as contact layers, (3) cleansing with mild antiseptics and application of an antiseptic cream (such as lipid-stabilized hydrogen peroxide) can reduce bacterial load, (4) topical antibiotics/antimicrobials (e.g. fusidic acid, mupirocin) should be used for short periods to prevent resistance and sensitization, (5) silver-containing products can be alternatively employed, paying attention to limit the time of administration and treated surface due to potential systemic absorption, (6) systemic antibiotics should be administered in the presence of multiple infected lesions and/or deep infection with surrounding tissue involvement.*

*STATEMENT 27—Healing of infected wounds should be closely monitored by clinical evaluation of wound (exudate) and perilesional area (erythema and swelling) appearance and odor, improvement of local symptoms (pain), and then size reduction.*

**4.3 Oral and dental care**

Oral blisters, ulcerations, inflammation and scarring often resulting in microstomia and ankyloglossia, as well as multiple caries cause discomfort and hinder feeding in most RDEB patients [9]. Therefore, oral hygiene and dental care are key though challenging aspects in RDEB management and should be performed by an experienced dentist [21, 23]. Principal measures for oral and dental care are:

- regular scheduling of visits,
- training of parents and patients to carry out all preventive measures (e.g. oral hygiene, fluoride use),
- personalized tailoring and practical demonstration of the tooth brushing technique, using small soft or electric brushes,
- administration of fluoride as a toothpaste or rinse solution,
- use of antiseptic mouthwashes twice a day (e.g. chlorhexidine 0.12%; polyhexanide 0.12%, without alcohol),
- regular professional tooth cleaning.

*STATEMENT 28—RDEB patients experience numerous oral problems: blisters, ulcerations, inflammation, and frequently severe scarring, as well as multiple caries causing pain and impairing feeding. Oral hygiene and dental care are important aspects of RDEB management.*

*STATEMENT 29—Preventive management should always be adopted before the teeth erupt; parents and patients should be trained to carry out all preventive measures (e.g. oral hygiene, fluoride), and visits should be regularly scheduled.*

*STATEMENT 30—Tooth brushing technique should be individually tailored and practically demonstrated. The use of small soft or electric brushes is recommended.*

*STATEMENT 31—Professional tooth cleaning should be regularly performed.*

**4.4 Gastrointestinal involvement**

In addition to oral involvement (see paragraph 4.3), RDEB gastrointestinal manifestations comprise esophageal strictures, gastroesophageal reflux disease (GERD), constipation, anal fissures and strictures [9, 41, 42].

Esophageal strictures are one of the commonest and most severe RDEB gastrointestinal complications, and can occur from early childhood [42, 43]. They cause chronic dysphagia (inability to swallow solid or even liquids, sialorrhea, regurgitation, and food impaction), and odynophagia, resulting in restricted food intake, and contributing to malnutrition and anemia. Prompt diagnosis and appropriate management of esophageal strictures are crucial aspects of RDEB care. The reference center gastroenterologist should evaluate the patients at first sign/symptom onset. In presence of suggestive clinical signs and symptoms, the diagnosis should be confirmed radiologically with contrast studies (esophagogram, videofluoroscopy), paying attention to the upper esophagus where strictures are more frequently located [43].

Non-pharmacological measures to prevent and delay progression of esophageal strictures comprise dietary modifications (e.g. soft, non-spicy food), and adequate oral and dental care [21, 39, 43]. Administration of oral viscous budesonide may be considered in symptomatic patients [22, 44, 45]. Esophageal dilation represents the first line treatment for esophageal strictures [43]. It should be performed in a reference centre, preferably by fluoroscopically-guided balloon dilation. Endoscopy-guided dilation represents an alternative option in particular for patients with gastrostomy in place or depending on reference centre resources and expertise. Stricture relapses are common and they can be treated by repeated dilations [43].

GERD should be regularly searched for in RDEB patients starting from infancy and, when present, treated with standard medical therapies [39, 41, 45]. Management of chronic constipation is discussed at paragraph 4.5.

*STATEMENT 32—Esophageal strictures are one of the most common and severe gastrointestinal complications, they can occur from early childhood. They cause chronic dysphagia and odynophagia, resulting in reduced food intake and malnutrition. Early diagnosis and appropriate management are crucial aspects of RDEB care.*

*STATEMENT 33—In patients with suggestive clinical signs and symptoms, esophageal stricture diagnosis should be confirmed radiologically with contrast studies (esophagogram, videofluoroscopy) paying attention to the upper esophagus where strictures are more frequently located.*

*STATEMENT 34—Non-pharmacological measures to prevent/delay progression of esophageal strictures comprise dietary modifications (soft, non-spicy food), and adequate dental and oral care.*

*STATEMENT 35—In patients with confirmed diagnosis, esophageal dilation should be performed in a reference center, preferably by fluoroscopically-guided balloon dilation. Stricture relapses are common and can be treated by repeated dilations.*

**4.5 Nutrition**

Maintenance of an adequate nutritional status is an essential and demanding issue from the first years of life in RDEB patients [9, 39, 46]. Key contributory factors to be addressed by the multidisciplinary team are an adequate skin and oral/dental care, as well as early diagnosis and treatment of gastrointestinal complications, and anaemia. The dietitian and nutritionist should be always involved in patient follow-up [39, 46].

Nutritional compromise occurs mainly in generalized RDEB, and depends on [39]:

- hypercatabolic inflammatory status, in which fluid and heat losses through wounds, increased protein turnover, and infections contribute to enhanced requirements. Thus, nutrient needs reflect the severity and extent of lesions,
- the degree to which oral complications (microstomia, ankyloglossia, caries), esophageal strictures, and other gastrointestinal complications limit food intake or affect absorption.

Nutritional support generally aims to (1) improve feeding and minimize nutritional deficiencies, (2) ameliorate growth, (3) optimize bowel function, (4) improve wound healing, and (5) promote pubertal development and sexual maturation [39].

In the absence of specific data in RDEB, best practice in designing nutrition support currently involves consideration of the following three main components:

1. Evaluation of factors affecting nutritional intake using a scoring system, such as «STRONGkids» [47].
2. Definition of energy requirements which can be estimated starting from those of age/height*—*and gender-matched unaffected children, with additional factors that consider the extent of skin lesions, presumed level of bacterial infection, and requirement for catch-up growth. Practically, the energy requirement usually ranges from 100 to 150% of the estimated average for normal children. Particularly elevated are protein requirements [46].
3. Evaluation of biochemical and hematological parameters to identify deficient micronutrients and vitamins that require supplementation, in particular zinc, selenium, vitamins C, 25(OH)D3, K, niacin, B6, and B12 [46, 48].

Iron deficiency is almost constant in these patients, and requires supplementation as described in paragraph 4.6.

Caloric intake should be increased gradually to avoid intolerance or even refeeding syndrome [39]. Meals should contain the highest caloric and nutrient content with the lowest possible volume. Commercial nutritional supplements with high energy and protein content are frequently required [46].

In RDEB children with insufficient growth, gastrostomy placement is recommended while continuing oral nutrition [39, 40].

Chronic constipation with painful defecation is a frequent complication of RDEB. Constipation should be promptly and regularly treated with increased fluid and fiber intake, and administration of macrogol (polyethylene glycol), when required [49].

*STATEMENT 36—The maintenance of an adequate nutritional status is extremely challenging in RDEB patients, and requires the constant involvement of a dietitian and a nutritionist in the follow-up of the patient.*

*STATEMENT 37—Nutritional support generally aims to: (1) improve feeding and minimize nutritional deficiencies, (2) ameliorate growth, (3) optimize bowel function, (4) improve wound healing, (5) promote pubertal development and sexual maturation.*

*STATEMENT 38—In the absence of specific data in RDEB, energy requirements may be estimated starting from those of age/height—and gender-matched unaffected children, with the addition of factors that consider the extent of skin lesions, presumed level of bacterial infection, and requirement for catchup growth. Practically, the energy requirement usually ranges from 100 to 150% of the estimated average for normal children.*

*STATEMENT 39—Biochemical and hematological parameters to identify deficient micronutrients and vitamins that require supplementation [in particular zinc, selenium, vitamins C, 25(OH)D3, K, niacin, B6, and B12] should be regularly evaluated.*

*STATEMENT 40—In RDEB children with insufficient growth, gastrostomy placement is recommended.*

*STATEMENT 41—Constipation should be promptly and regularly treated with increased fluid and fiber intake, and administration of macrogol (polyethylene glycol), when required.*

**4.6 Anemia**

Anemia is a common complication of RDEB [48, 50]. It can manifest already in the first year of life [50]. The etiology is multifactorial, with iron deficiency and chronic inflammation being the primary factors. Iron deficiency is due to iron losses from chronic bleeding wounds, and poor dietary intake and absorption. Early diagnosis and management of iron deficiency and anemia are crucial to decrease anemia-related symptoms, promote wound healing, increase growth, and improve QoL [28]. For generalized RDEB forms, anemia should be evaluated twice a year starting from infancy [28]. The diagnosis should be based on the WHO recommendations and requires a careful clinical examination and history, taking into account dietary intake, oral and gastrointestinal involvement, wound extent and chronic bleeding, recurrent infections, as well as recent surgery [28]. The gold standard for diagnosis of anemia is hemoglobin (Hb) level. In addition to complete blood and reticulocyte count and C-reactive protein, iron profile including serum iron, ferritin, total iron-binding capacity (TIBC), and transferrin saturation should be performed. Low ferritin indicates iron deficiency, while normal ferritin does not exclude it, as ferritin can be increased in acute and chronic inflammatory conditions (thus masking iron deficiency). An elevated TIBC is a marker of iron deficiency [28].

Treatment should be individualized; it includes dietary measures, iron supplementation and blood transfusion. Oral iron can be administered in mild anemia (Hb levels > 10 g/dL); iron infusion is reserved for moderate to severe anemia (Hb level < 10 g/dL), or in patients who do not tolerate oral iron. Blood transfusion should be performed if Hb is < 8 g/dL in adults and < 6 g/dL in children [28].

*STATEMENT 42—Anemia is a common complication of RDEB. The etiology is multifactorial, with iron deficiency and chronic inflammation being the primary factors. Iron deficiency is due to iron losses from chronic bleeding wounds, and poor dietary intake and absorption. Early diagnosis and management of iron deficiency and anemia are crucial to decrease anemia-related symptoms, promote wound healing, increase growth, and improve quality of life.*

*STATEMENT 43—For generalized RDEB forms, anemia should be evaluated twice a year starting from infancy.*

*STATEMENT 44—The gold standard for diagnosis of anemia is hemoglobin (Hb) level. Diagnosis and severity of anemia will be based on the WHO recommendations. Low ferritin level and elevated total iron-binding capacity (TIBC) can support diagnosis of iron deficiency.*

*STATEMENT 45—Treatment includes dietary measures, iron supplementation and blood transfusion. Oral iron can be administered in mild anemia (Hb levels* > *10 g/dL); iron infusion is reserved for moderate to severe anemia (Hb level* < *10 g/dL), or in patients who do not tolerate oral iron. Blood transfusion should be performed if Hb is* < *8 g/dL in adults and* < *6 g/dL in children.*

**4.7 Ocular involvement**

Ocular manifestations affecting the conjunctiva, cornea and eyelids are common in RDEB (> 50% of patients) and can present from the first months of life [9, 51]. They comprise chronic blepharitis, eyelid blisters, recurrent corneal erosions that can lead to scarring, opacities, impaired vision and, rarely, blindness. The main symptoms are red watering eyes, photophobia and ocular pain [51].

All patients should be referred to the ophthalmologist of a reference center for a baseline examination and should be followed as frequently as deemed necessary according to the severity of ocular findings [51].

Secondary corneal dryness is treated with sterile ophthalmologic lubricating ointments and preservative-free artificial tears. Practical measures to reduce tear film evaporation include bedroom humidifiers [51]. Bandage contact lens associated with antibiotic prophylaxis can be considered to treat recurrent corneal erosions affecting visual acuity [49]. Eyelash collarettes should be treated with topical fusidic acid ointment, avoiding lid scrubs, which can cause lid blistering in RDEB patients [51].

*STATEMENT 46—Ocular manifestations affecting the conjunctiva, cornea and eyelids are common in RDEB (*> *50% of patients). They comprise chronic blepharitis, eyelid blisters, recurrent painful corneal erosions leading to scarring, opacities, impaired vision and, rarely, blindness. All patients should be referred to the ophthalmologist of a reference center for a baseline examination and should be followed as frequently as deemed necessary according to the severity of ocular findings.*

*STATEMENT 47—Secondary corneal dryness is treated with sterile ophthalmologic lubricating ointments and preservative-free artificial tears. Practical measures to reduce tear film evaporation include bedroom humidifiers.*

**4.8 Hand and foot deformities**

In RDEB patients, hands and feet are particularly prone to repeated blistering, ulceration and scarring. Hand deformities comprise thumb adduction contractures, digit pseudosyndactyly, and flexion contractures of all joints including the wrist. They are almost constant in severe RDEB, and result in major functional impairment. Thus, a regular management is required [20, 26].

Members of the multidisciplinary team involved in hand deformity prevention and management are the dermatologist, hand or plastic surgeon, anesthetist, occupational therapist, and physiotherapist.

Treatments are aimed at: (1) delaying contractures and deformities with medical and occupational therapy and physiotherapy (see paragraph 5.3), (2) improving function with surgery [20, 26], and (3) delaying recurrence after surgery with splinting and meticulous skin care and physiotherapy [26].

Surgical hand techniques include either releasing the thumb only, with restoration of prehension and grasping, or whole hand and pseudosyndactyly release. Functional improvement with surgery is always temporary with recurrences occurring within 1–2 years. Before any surgery is planned, the patient and family members must be fully informed on surgical procedure, complications (pain, bleeding, and infections) and constant relapses, and necessity of hand physiotherapy and splinting to delay relapse after surgery [26].

In addition to blistering and scars, RDEB podiatric manifestations include nail dystrophy and structural abnormalities/deformities affecting foot positioning [20]. Generally, the management tends to be supportive and aimed to prevent blistering by providing information on suitable shoes, cushioning materials, and appropriate insoles or orthotics, and to educate to nail care by regular trimming [20]. Physiotherapy and occupational therapy are crucial to improve and maintain mobility (see paragraph 5.3).

*STATEMENT 48—In RDEB patients, hands and feet are particularly prone to repeated blistering, ulceration and scarring. Hand deformities comprise thumb adduction contractures, digit pseudosyndactyly, and flexion contractures of all joints including the wrist. They are almost constant in severe RDEB, and result in major functional impairment. Thus, a continuous management is required.*

*STATEMENT 49—Treatments are aimed at delaying deformities and contractures with medical and occupational therapy/physiotherapy; improving function with surgery; delaying recurrence with splinting and meticulous skin care.*

*STATEMENT 50—Hand surgery results in functional improvement which is always temporary. Before any surgery is planned, the patient and family members must be fully informed on surgical procedure, complications (pain, bleeding, infections) and constant relapses, and necessity of hand physiotherapy and splinting to delay relapse after surgery.*

*STATEMENT 51—In addition to blistering and scars, RDEB podiatric manifestations include nail dystrophy and structural abnormalities/deformities affecting foot positioning. Generally, the management tends to be supportive and aims to prevent blistering by providing information on suitable shoes, cushioning materials, and appropriate insoles or orthotics. Physiotherapy and occupational therapy are crucial to improve and maintain mobility.*

**4.9 Cutaneous squamous cell carcinoma**

All RDEB subtypes are associated with an increased risk of developing cSCC. This epithelial skin cancer is characterized by a rapid growth and an aggressive biological behaviour and represents the first cause of mortality in RDEB patients [17].

Despite extensive research, knowledge of RDEB-cSCC etiopathogenesis remains limited [7, 10]. Genetic studies disclosed a mutation signature consistent with mutagenesis related to the increased enzymatic activity of apolipoprotein B mRNA editing enzyme, catalytic polypeptide-like (APOBEC) [7, 10]. However, the genetic determinants of RDEB-cSCC so far identified do not explain their aggressive behavior and increased metastatic potential. Current evidence points to a key role of the inflammatory, infection-prone and fibrotic microenvironment combined with an altered host immune response in the development and aggressiveness of RDEB-cSCC [7, 10, 52].

Clinical diagnosis of cSCC may be difficult as this cancer usually develops on chronic wounds. Suggestive clinical features are [17]:

- non-healing chronic wounds despite adequate treatment,
- rapid wound enlargement,
- deep wound with raised or rolled edges,
- exuberant/vegetating wound appearance,
- areas of thick hyperkeratosis,
- increased wound pain or a tingling sensation.

The time frame for considering a wound as non-healing varies based on patient’s age and clinical features, and wound site and should be evaluated taking into account the healing time of “normal” wounds in the same patient.

Development of cSCC may occur starting from the second decade of life in patients with severe RDEB. Thus, skin total body examination should be performed by an expert dermatologist from an EB reference center every 3–6 months starting from 9 to 10 years of age. Individuals with a history of cSCC should be evaluated at 3-month intervals. Patients and families should be educated about the risk of cSCC and clinical features and symptoms suggestive for wound malignant transformation. Diagnostic biopsies for histopathological examination should always be performed in clinically suspicious areas. To reduce the risk of misdiagnosis, multiple biopsies should be taken [17]. Histopathological examination is better performed by a pathologist from an EB reference center. Indeed, differential diagnosis between cSCC and granulation tissue or pseudoepitheliomatous hyperplasia can be challenging in RDEB [10, 17].

Wide surgical excision remains the first-line treatment for RDEB cSCC [17]. The surgical approach is defined by the surgeon/plastic surgeon, in collaboration with the dermatologist and oncologist, taking into account the anatomical location and size of the lesion, RDEB skin fragility, as well as patient preference [17]. Alternative therapeutic options including radiotherapy, chemotherapy, electrochemotherapy or targeted therapy with EGFR inhibitors [17, 53] should be considered when surgical excision is not feasible, and in locally advanced or metastatic disease. At present, immunotherapy with programmed cell death protein 1 inhibitors is approved for metastatic and locally advanced cSCC and has been employed in a limited number of RDEB patients, providing some clinical benefit [54, 55].

The patient and family should be fully informed about surgical techniques, possible alternative treatments, expected results, as well as consequences on functionality and ability to carry out daily activities [17].

*STATEMENT 52—All RDEB subtypes are associated with an increased risk of developing cutaneous squamous cell carcinomas (cSCCs). This epithelial skin cancer is characterized by a rapid growth and an aggressive biological behavior and represents the first cause of mortality in RDEB patients. Early cSCC diagnosis is a crucial aspect of RDEB care.*

*STATEMENT 53—Clinical diagnosis of cSCC may be difficult as this cancer usually develops on chronic wounds. Suggestive clinical features are: (1) non-healing chronic wounds despite adequate treatment, (2) rapid wound enlargement, (3) deep wound with raised or rolled edges, (4) exuberant/vegetating appearance, (5) areas of thick hyperkeratosis, and (6) increased wound pain or a tingling sensation.*

*STATEMENT 54—Development of cSCC may occur starting from the second decade of life in patients with severe RDEB. Thus, total body skin examination should be performed by an expert dermatologist from an EB reference center every 3–6 months starting from 9 to 10 years of age. Patients with a history of cSCC should be evaluated at 3-month intervals.*

*STATEMENT 55—Diagnostic biopsies for histopathological examination should always be performed in suspicious areas. To reduce the risk of misdiagnosis, multiple biopsies should be taken.*

*STATEMENT 56—Wide surgical excision is the first-line treatment for RDEB cSCC. The surgical approach is defined by the surgeon/plastic surgeon, in collaboration with the dermatologist and oncologist, taking into account the anatomical location and size of the lesion, RDEB skin fragility, as well as patient preference.*

*STATEMENT 57—Alternative therapeutic options including radiotherapy, chemotherapy, electrochemotherapy or targeted therapy with EGFR inhibitors should be considered when surgical excision is not feasible, and in locally advanced or metastatic disease. At present, immunotherapy with programmed cell death protein 1 inhibitors is approved for metastatic and locally advanced cSCC. It has been employed in a limited number of RDEB patients providing some clinical benefit.*

**4.10 Delayed puberty and osteoporosis**

Recent studies have highlighted a relevant prevalence of delayed puberty in RDEB, involving one third to a half of patients [56, 57]. The main causes seem to be malnutrition and low body weight resulting in a delay in hypothalamic-pituitary–gonadal axis maturation, associated to chronic inflammation [56]. Due to the permissive effects of sex hormones on mineralization, pubertal delay can negatively affect peak bone mass achievement [56, 57]. Bone impairment correlates with disease severity and skin damage [56]. Additional contributory factors for osteopenia/osteoporosis are malnutrition, with a consequent calcium deficiency, impaired mobility, and hypovitaminosis D also due to limited sun exposure [58, 59]. Low bone mineral density is reported in about 30% of RDEB pediatric patients, occurring frequently prior to 10 years of age and further declining during adolescence [57]. Osteoporosis has been reported in 75% of severe RDEB adults [58]. From 9 to 10 ears of age, RDEB patients should be regularly evaluated by the endocrinologist at the reference center to early detect bone mineralization defects and pubertal delay. In particular, evaluation of bone mineral density should be considered starting from adolescence in patients with severe RDEB [58].

Preventive measures should be taken from early childhood to favor bone growth and prevent osteopenia/osteoporosis. Adequate nutrition, calcium and vitamin D supplementation as well as physiotherapy are recommended [58, 59]. Despite lack of specific literature data, it might be useful to treat the delay of puberty with sex hormone supplementation.

*STATEMENT 58—Pubertal delay is highly prevalent in RDEB, involving one third to a half of patients, and being mainly related to malnutrition and inflammatory status. From 9 to 10 years of age, RDEB patients should be regularly evaluated by the endocrinologist at the reference center to early detect delay of puberty.*

*STATEMENT 59—Low bone mineral density is reported in about 30% of RDEB pediatric patients. Osteoporosis has been detected in 75% of severe RDEB adults. Evaluation of bone mineral density should be considered starting from adolescence in patients with severe RDEB.*

*STATEMENT 60—Preventive measures should be taken from early childhood to favor bone growth and prevent osteopenia/osteoporosis. Adequate nutrition, calcium and vitamin D supplementation as well as physiotherapy are recommended.*

**4.11 Renal involvement**

RDEB patients may develop renal involvement, which can progress to renal failure, leading to a significant increase of morbidity and even mortality [9, 60, 61]. IgA nephropathy, post-infectious glomerulonephritis, glomerulonephritis with C3 deposition, proteinuria or nephrotic syndrome secondary to amyloidosis, and focal segmental glomerulosclerosis are reported [60, 61].

Despite data paucity, there is a general consensus that renal function should be monitored early and regularly, as immunosuppressive treatment can be considered in some glomerulonephritis forms. Renal function should be assessed by measurement of creatinine, urinalysis and blood pressure every 6–12 months. In case of abnormal findings, the nephrologist should be promptly involved. As RDEB patients are often malnourished, plasma creatinine could overestimate the real renal function. In malnourished subjects, nephrologists suggest that cystatin C is a more accurate marker of renal function than serum creatinine [60, 61].

When dialysis is required, dermatologists and nephrologists should evaluate case-by-case the most suitable modality: hemodialysis and peritoneal dialysis are both reported in RDEB patients [60, 61].

*STATEMENT 61—RDEB patients may develop renal involvement, which can progress to renal failure, leading to a significant increase of morbidity and even mortality. Evaluation of renal function should be performed every 6–12 months as part of RDEB patient follow-up. We suggest to evaluate plasma creatinine (and cystatin C in case of malnutrition), urinalysis and blood pressure. In case of abnormal findings, nephrologist should be promptly involved.*

*STATEMENT 62—Hemodialysis and peritoneal dialysis are both reported in RDEB patients. Dermatologists and nephrologists should evaluate case-by-case the most suitable dialysis modality, when needed.*

**4.12 Sexuality, pregnancy and delivery**

RDEB manifestations and complications have a high impact on sexual life. Multidisciplinary team members should consider and address psychosocial and medical issues related to sexuality and pubertal/sexual development [24, 27].

Pregnancy has been reported in women with RDEB. However, nutritional compromise, low body mass index, and delayed puberty may affect fertility in these patients. In addition, mucosal fragility may impact on sexual function [27]. Pre-pregnancy diet and nutrition optimization may improve maternal and perinatal outcomes. In addition, pregnancy-related nausea and vomiting should be treated in particular in patients with pre-existing GERD and/or esophageal strictures [27]. The multidisciplinary team members who should be early involved in management of pregnancy and delivery are the obstetrician, midwife, anesthetist, clinical nurse specialist, dermatologist, nutritionist, and psychologist [27]. Although RDEB is not an absolute contraindication to vaginal birth, an individualized birth plan should be discussed among the team members. Instrumental delivery, including vacuum suction or forceps-assisted delivery should be avoided, whenever possible [27].

*STATEMENT 63—RDEB manifestations and complications have a high impact on sexual life. Multidisciplinary team members should consider and address psychosocial and medical issues related to sexuality and pubertal/sexual development.*

*STATEMENT 64—Pregnancy has been described in women with RDEB. However, nutritional compromise, low body mass index, and delayed puberty may affect fertility in these patients. Pre-pregnancy diet and nutrition optimization may improve maternal and perinatal outcomes.*

*STATEMENT 65—The multidisciplinary team members who should be early involved in management of pregnancy and delivery are obstetrician, midwife, anesthetist, clinical nurse specialist, dermatologist, nutritionist, and psychologist.*

*STATEMENT 66—Although RDEB is not an absolute contraindication to vaginal birth, an individualized birth plan should be discussed among the multidisciplinary team members. Instrumental delivery, including vacuum suction or forceps-assisted delivery should be avoided, whenever possible.*

**5. Transversal age-independent issues**

**5.1 Pain and itch management**

Pain is one of the most common and disabling symptoms in RDEB patients starting from the first days of life. It is primarily related to mucocutaneous wounds, but also to various disease complications (e.g. GERD, constipation, joint contractures). Patients suffer from acute and chronic pain that exacerbates during each procedure (e.g. bathing, wound dressing), and need adequate treatment [16, 23]. RDEB pain is nociceptive, neuropathic, and psychogenic. Pain memory in children leads to increased future pain intensity fear and distress. Thus, pain should be promptly assessed and treated by the reference centre pain therapist [23].

For pain assessment, visual analogue (VAS) or numeric rating scales (NRS) should be used from 7 years of age. For younger children, a behavioural scale (FLACC, face, legs, activity, cry, consolability) can be employed. Moreover, it is essential to properly assess the type of pain (nociceptive, neuropathic) and the level of anxiety of the patient in order to optimize treatment [23].

For mild pain (pain-NRS or FLACC < 4/10), non-opioid analgesics (e.g. acetaminophen, ibuprofen) are recommended. Moderate or severe pain requires an opioid analgesic (e.g. nefopam, tramadol, morphine, oxycodone, methadone). Opioid dosage should be increased as required to achieve an effective analgesia in patients developing tolerance as a consequence of chronic administration. Possible side effects of opioids, in particular itch worsening and constipation, should be considered. Tricyclic antidepressants (e.g. amitriptyline) or anti-epileptics (e.g. gabapentin) can be associated for chronic pain [23]. Recently, combined tetrahydrocannabinol and cannabidiol have been used to treat pain in EB [23]. Additional analgesia should be regularly administered before dressing changes which should be carried out in a relaxing context [16, 23].

Non-pharmacological and psychological therapies, including cognitive behavioural therapy, hypnosis, biofeedback and relaxation techniques, may all contribute to reduce pain intensity and related distress, and to improve pain coping and QoL [16, 23].

Itch, usually chronic, is also a common symptom, with a major impact on QoL. It occurs more frequently at wound sites, but may also be generalized, and should be adequately managed. Possible triggering factors include skin dryness, wound healing process and associated inflammation, wound dressings, opioids, and stress. Pruritus triggers a vicious cycle of itch-scratching that causes new blisters and worsens wounds [62].

Topical therapies comprise bathing in tepid water with syndet/oil cleanser, skin hydration with emollients, and short courses of corticosteroids. Widely used systemic treatments are sedating and non-sedating antihistamines, and also tricyclic antidepressants (amitriptyline, doxepin) and anticonvulsants (gabapentin, pregabalin). However, results are often unsatisfactory. Other medications reported in single cases/case series include antipsychotic agents (olanzapine), serotonin reuptake inhibitors (fluoxetine, paroxetine, serlopitant), opioid antagonists (naloxone, naltrexone), cannabinoids, and anti-inflammatory agents (cyclosporine, thalidomide). Efficacy of the anti-IL-4R-alfa monoclonal antibody dupilumab has been recently described in several patients, in particular affected by DEB pruriginosa [62, 63].

The use of non-pharmacological interventions, such as cognitive behavioural therapy, yoga, hypnosis, and support groups, has also been reported in small case series [62].

*STATEMENT 67—Pain is one of the most common and disabling symptoms in RDEB patients starting from the first days of life. Pain is primarily related to mucocutaneous wounds, but also to different disease complications (e.g. GERD, constipation, joint contractures). Patients suffer from acute and chronic pain that exacerbates during each procedure (e.g. bathing, wound dressing), and need adequate treatment.*

*STATEMENT 68—RDEB patient pain is nociceptive, neuropathic, and psychogenic. Pain memory in children leads to increased future pain intensity fear and distress. Thus, pain should be promptly assessed and treated by the pain therapist.*

*STATEMENT 69—For mild pain (pain-NRS or FLACC* < *4/10), non-opioid analgesics (e.g. acetaminophen, ibuprofen) can be used. Moderate or severe pain requires an opioid analgesic (e.g. nefopam, tramadol, morphine, oxycodone, methadone). Tricyclic antidepressants (e.g. amitriptyline) or anti-epileptics (e.g. gabapentin) can be associated for chronic pain.*

*STATEMENT 70—Non-pharmacological and psychological therapies, including cognitive behavioural therapy, hypnosis, biofeedback and relaxation techniques, may all contribute to reduce pain intensity and related distress, and to improve pain coping and quality of life.*

*STATEMENT 71—Additional analgesia should be regularly administered before dressing changes which should be carried out in a relaxing context.*

*STATEMENT 72—Itch (pruritus), usually chronic, is also a common symptom, with a major impact on quality of life. It occurs more frequently at wound sites but may also be generalized, and should be adequately managed.*

*STATEMENT 73—Topical therapies comprise bathing in tepid water with syndet/oil cleanser, skin hydration with emollients, and short courses of corticosteroids. Widely used systemic treatments are sedating and non-sedating antihistamines, tricyclic antidepressants (amitriptyline, doxepin) and anticonvulsant (gabapentin, pregabalin).*

**5.2 Patient care in the operating theatre**

In RDEB patients, surgery in sedation/general anaesthesia should be limited to strictly necessary procedures following discussion among the involved multidisciplinary team members. A multidisciplinary re-assessment, including specialist anesthetic evaluation, should be performed a week or two prior to the surgical procedure to verify patient general conditions, in particular anemia and possible infections [15, 64].

Specific measures should be regularly implemented in order to reduce trauma:

- sedative premedication to prevent excessive patient movement and subsequent blisters [64],
- appropriate training of the anesthesiology team to avoid skin and mucosal rubbing prior to and during surgery (e.g. padding of frictional/pressure areas, avoidance of all adhesive materials, patient moving using a blanket, surgical site disinfection by solution pouring or gentle dabbing, lubrication of all airway equipment) [64],
- adequate equipment and materials available in the theatre (e.g. antidecubitus mattress, non–adherent silicon-based dressings and tapes, clip-type pulse oximetry) [15].

RDEB patient airway management can be particularly challenging due to microstomia and oro-pharyngeal scarring: in these cases, the Ear Nose and Throat specialist should be part of the anesthesiology team [21, 64].

After surgery, the intravenous line should be kept in place as long as possible to perform transfusion, perfusion or other systemic therapy (e.g. iron, albumin, antibiotics) if needed [15].

*STATEMENT 74—In RDEB patients, surgery in sedation/general anesthesia should be limited to strictly necessary procedures following discussion among multidisciplinary team members.*

*STATEMENT 75—The anesthetic team should be adequately trained to avoid rubbing or stroking patient skin and mucosae prior to and during surgery (e.g. padding of frictional/pressure areas, avoidance of all adhesive materials, patient moving using a blanket, surgical site disinfection by solution pouring or gentle dabbing, lubrication of all airway equipment). In parallel, the operating theatre should be adequately equipped (e.g. antidecubitus mattress, non-adherent silicon-based dressings and tapes, clip-type pulse oximetry).*

*STATEMENT 76—RDEB patient airway management can be particularly challenging due to microstomia and oro-pharyngeal scarring: in these cases, the Ear Nose and Throat specialist should be included in the anesthetic team.*

**5.3 Physiotherapy and occupational therapy**

Due to fibrosis, scarring, joint contractures and pain, RDEB patients can manifest reduced functional mobility and limited motor skills, with a major impact on daily life activities. An essential component of the interdisciplinary team is the reference center physiotherapist [25].

Physiotherapy should aim to optimize [25]:

- developmental motor milestone attainment,
- safe and functional mobility,
- ambulation endurance,
- ability to safely bear weight,
- interaction with environment.

In addition to the physiotherapist, an occupational therapist may be involved in RDEB patient care with the aim to optimize independence and preserve QoL. Occupational therapy interventions may help improving patient abilities in all activities of daily life, hand function, fine motor development and retention, as well as oral feeding skills [18].

*STATEMENT 77—Due to fibrosis, scarring, joint contractures and pain, RDEB patients can manifest reduced functional mobility and limited motor skills, with a major impact on daily life. The physiotherapist should be early involved.*

*STATEMENT 78—The physiotherapist should assess functional ability and aim to optimize: (1) developmental motor milestone attainment, (2) safe and functional mobility, (3) ambulation endurance, (4) ability to safely bear weight, and (5) interaction with environment.*

*STATEMENT 79—In addition to the physiotherapist, an occupational therapist may be involved in RDEB patient care with the aim to optimize independence and preserve quality of life.*

*STATEMENT 80—Occupational therapy interventions may help improving patients' abilities in daily activities, hand function, fine motor development and retention, as well as oral feeding skills.*

**5.4 Therapeutic patient education**

Therapeutic patient education (TPE) is an ongoing, patient-centered procedure that helps families and patients with chronic illnesses better manage their health and, in general, increase treatment compliance. The procedure is dynamic and should consider the patient age, clinical characteristics and complications during disease course, and the ensuing psychological effects. It should be also adapted to the family/patient socio-cultural level [35].

Considering the neonatal onset of RDEB, its management complexity and familial psychological implications, TPE should be delivered to the parents starting from the earliest stages of life following the development of a positive relationship between them and the members of the multidisciplinary team [35]. Before infant discharge, parents/caregivers should be clearly informed about RDEB course and trained to take care of all disease aspects from skin and wound management until nutrition. During subsequent follow-ups, TPE should be periodically checked and updated with the involvement of the dermatologist and specialized nurse for wound and skin care, which represents the most complex and time-consuming procedure of disease management. The pediatrician and nutritionist/dietitian will be involved for nutritional aspects, and other specialists could be implicated overtime depending on disease manifestations and complications. The support of a psychologist in delivering TPE is desirable [35].

From roughly the age of five, the child should directly get age-appropriate TPE. In adolescent patients, treatment compliance typically declines, while additional severe complications frequently manifest (e.g. cSCC development, renal failure), making TPE more challenging [35].

*STATEMENT 81—Therapeutic patient education (TPE) is a continuous process of patient-centered medical care, enabling patients affected by chronic diseases, and their families, to better manage their illness and, overall, improving adherence to treatment. The process is dynamic: it should consider patient age, modification of clinical features and complications in disease course, and consequent psychological impact.*

*STATEMENT 82—Before infant discharge, parents/caregivers should be clearly informed about RDEB and trained to take care of all disease aspects from wound management to nutrition.*

*STATEMENT 83—During subsequent follow-ups, TPE should be periodically checked and updated with the involvement of the dermatologist and specialized nurse for wound and skin care, which represents the most complex and time-consuming aspect of disease management, as well as of the pediatrician and nutritionist/dietitian for nutrition. The support of a psychologist in delivering TPE is desirable. Other specialists will be involved overtime depending on disease manifestations and complications.*

*STATEMENT 84—Age-appropriated TPE should be delivered also to the child starting from about 5 years of age. During adolescence, compliance to treatment usually decreases, while complications increase (including the risk of developing cSCC), making TPE more challenging but also more essential.*

**5.5 Psychosocial support**

RDEB has a profound impact on all domains of patient and family life, including social interactions, education, employment, and leisure. Thus, psychosocial support should be guaranteed to patients lifelong, in order to improve their QoL and well-being, and to help them coping with the disease [19]. Psychosocial care for family and caregivers is also recommended to ameliorate their QoL and well-being and to prevent family breakdown [19].

Finally, the burden of RDEB can also affect healthcare professionals, who can benefit from working in teams where they have access to shared support and can discuss difficulties [19].

*STATEMENT 85—RDEB has a profound impact on all domains of patient and family life, including social interactions, education, employment and leisure. Thus, lifelong psychosocial support should be guaranteed to patients, in order to improve their quality of life (QoL) and well-being, and to help them coping with the disease.*

*STATEMENT 86—Psychosocial care for family and caregivers is also recommended to improve their QoL and well-being and to prevent family breakdown.*

## Discussion

RDEB is a rare, severe and highly disabling disease directly or indirectly affecting many organs, with a reduced life expectancy and a heavy impact on patient QoL and their families. Management of the clinical manifestations requires an integrated and coordinated multidisciplinary approach that should be continuously adapted based on patient age and complications. Existing diagnostic or therapeutic recommendations generally focus on single aspects or specific ages of EB patient management, making it difficult for clinicians to have a unique and comprehensive reference. The present study offers a series of practical and synthetic recommendation statements covering all major issues in the management of patients with RDEB from birth to adulthood. Thus, it can represent a useful and practical tool for healthcare personnel working in reference centers as well as in community hospitals and primary care.

However, the present study has several limitations: first, the use of a consensus method may limit the value of the statements, which also reflect the opinion and expertise of the involved clinicians on the proposed issues. However, statement development by expert panel members was based on an updated literature search, comprising available guidelines, recommendations, and randomized trials, as well as retrospective case series. In addition, the high degree of consensus obtained by the multidisciplinary Delphi Study Group members for all topics reflects a uniform evidence-based approach to the management of RDEB patients by Italian clinicians. Another limitation of the study may derive from the fact that only one voting round was performed. However, since a full agreement was reached for all statements after the first round, it was estimated that further rounds would not have substantially modified the overall results of the study. A third limitation is that only Italian experts were involved. Nevertheless, most reference centers participating to this consensus are members of ERN-Skin and implicated in recommendation development on different aspects of rare skin diseases. Finally, the consensus development has contributed to reinforce the existing network at the national level and will hopefully favor further collaborations.

In conclusion, the present recommendations are expected to support clinical decision making in the complex multidisciplinary management of RDEB individuals throughout their life.

## Supplementary Information

**Additional file 1.**

## Data Availability

Results of the voting phase of the Delphi protocol are available on request.
