# Supplementary material for: Taking care of patients with recessive dystrophic epidermolysis bullosa from birth to adulthood: a multidisciplinary Italian Delphi consensus
Source: Orphanet J Rare Dis. 2025 Mar 16;20:128. doi: 10.1186/s13023-025-03635-1 (PMC11912768; doi:10.1186/s13023-025-03635-1)
Supplement: Supplementary file 1 — Additional file 1. [file 13023_2025_3635_MOESM1_ESM.docx]

**Table S1.** Voting results obtained from the Delphi process for the whole set of statements (number of respondents = 30)

| **Statements** | **Voting results** | | | | | |
| --- | --- | --- | --- | --- | --- | --- |
| **1. GENERAL PREMISE** | **%**  **strongly agree (score 5)** | **%**  **agree**  **(score 4)** | **% undecided**  **(score 3)** | **%**  **disagree**  **(score 2)** | **%**  **strongly disagree**  **(score 1)** | **Median score**  **(IQR)** |
| 1. Care of RDEB patients, from diagnosis to treatment, should be performed in reference centers offering coordinated multidisciplinary management by trained personnel. | 90 | 10 | 0 | 0 | 0 | 5 (1) |
| **2. DIAGNOSIS** |  | | | |  |  |
| 2. Diagnosis must be performed as early as possible in order to initiate the most appropriate treatment. | 87 | 13 | 0 | 0 | 0 | 5 (1) |
| 3. The diagnosis of RDEB is based on a combination of clinical features, family history and laboratory findings, including immunofluorescence antigen mapping on a skin biopsy and molecular genetic testing. | 74 | 23 | 3 | 0 | 0 | 5 (2) |
| 4. Genetic testing enables genetic counselling and DNA-based prenatal diagnosis. | 57 | 40 | 3 | 0 | 0 | 5 (2) |
| 5. The communication of the diagnosis should involve the dermatologist, the neonatologist/pediatrician and, for genetic diagnosis, the medical geneticist. It should be addressed to both parents, adapting the information to the family socio-cultural level. A psychologist should support the family. | 87 | 13 | 0 | 0 | 0 | 5 (1) |
| **3. NEONATAL AGE AND INFANCY** |  | | | |  |  |
| **3.1 Management of RDEB newborns and infants: general measures** |  | | | | | |
| 6. Members of the multidisciplinary team usually involved in RDEB care in infancy are neonatologist/pediatrician, dermatologist, nutritionist/dietitian, anesthesiologist, ophthalmologist, dentist, psychologist and specialized nurses. Parents should be gradually and regularly trained in the care of their child. | 77 | 20 | 3 | 0 | 0 | 5 (2) |
| 7. In RDEB newborns/infants, all cautions should be taken to reduce friction risk and minimize neonate/infants handling (lift the baby on a mattress and avoid sliding, choose appropriate garments, etc.); specific measures should be applied for routine interventional procedures (e.g. limit incubator use, avoid adhesive tapes, secure small electrodes with non-adhesive dressings, thick padding below the blood pressure cuff, whenever possible avoid naso- and oro-pharyngeal suction). | 80 | 20 | 0 | 0 | 0 | 5 (1) |
| 8. In severely affected RDEB newborns, (i) blood sampling for hematology and biochemistry tests, and (ii) swabs of suspicious/infected wounds for culture should be performed, and (iii) a venous access should be guaranteed. | 63 | 37 | 0 | 0 | 0 | 5 (1) |
| **3.2 Management of RDEB newborns and infants: skin care** |  | | | | | |
| 9. Adequate analgesia must be administered before bathing and wound care. | 67 | 33 | 0 | 0 | 0 | 5 (1) |
| 10. The diaper area requires specific protective (lining of disposable nappies with soft material) and cleansing measures (e.g. with liquid and white soft paraffin in equal parts or with an emollient/oil-based cleanser). Wounds should be managed according to principles applied for other body areas; however, paraffin-impregnated gauzes represent a cost-effective alternative. | 53 | 47 | 0 | 0 | 0 | 5 (1) |
| 11. Particular attention should be paid to hand and foot wound dressing from the first days of life to delay digit fusions. | 67 | 33 | 0 | 0 | 0 | 5 (1) |
| **3.3 Management of RDEB newborns and infants: feeding, discharge and follow-up** |  | | | | | |
| 12. Breastfeeding is encouraged: appropriate measures should be taken to reduce friction from rooting reflex (lubricate nipple and breast as well as infant lips and face). For oral feeding, commercially available teats should be adapted (enlarged or extra holes, softening with warm water) to facilitate sucking. | 60 | 40 | 0 | 0 | 0 | 5 (1) |
| 13. Whenever possible nasogastric feeding should be avoided. If required, it can be employed only in the short-term using a small soft lubricated polyurethane tube. In severe cases, gastrostomy should be considered to ensure adequate nutritional status since infancy. | 53 | 47 | 0 | 0 | 0 | 5 (1) |
| 14. The infant should be discharged from the hospital when the general health condition is stable, and the parents are adequately trained and confident in caring for their baby. | 73 | 27 | 0 | 0 | 0 | 5 (1) |
| 15. A first follow-up visit with the specialized team (usually: dermatologist, pediatrician, EB nurse, psychologist) should be organized in 2-4 weeks. Next appointment will usually be after one month, then every three months during infancy. | 54 | 43 | 3 | 0 | 0 | 5 (2) |
| **4. FROM CHILDHOOD TO ADULTHOOD** |  | | | | | |
| **4.1 Follow-up: general measures and timing** |  | | | | | |
| 16. After infancy, follow-up should be scheduled at least every 6 months. It can be more frequent depending on specific needs and disease complications. | 67 | 33 | 0 | 0 | 0 | 5 (2) |
| 17. Each follow-up visit should include evaluation of skin and mucosal involvement, general health status, adherence to treatment, and disease complications (e.g. chronic wounds, chronic pain/itching, dysphagia, anemia/malnutrition) and should be performed in the context of a multidisciplinary team including a dermatologist, pediatrician/internist, dentist, nutritionist, dietitian, specialized nurse, and physiotherapist. Additional specialists who may be involved on a case-by-case basis are ophthalmologist, pain therapist, anesthesiologist, interventional radiologist, pediatric digestive surgeon, gastroenterologist, oncologist, endocrinologist, cardiologist, nephrologist, orthopedist, plastic surgeon, Ear, Nose and Throat (ENT) specialist, and speech therapist. Psychological problems and social relationships should also be evaluated with the support of the psychologist and social worker. | 74 | 23 | 3 | 0 | 0 | 5 (2) |
| 18. Immunization schedule for infectious diseases should be regularly continued. | 57 | 40 | 3 | 0 | 0 | 5 (1) |
| 19. Particular attention should be given at the transition from the pediatric to the adult reference center, in order to warrant the continuity of care and possibly preserve patients’ quality of life. | 77 | 23 | 0 | 0 | 0 | 5 (1) |
| **4.2 Wound care** |  | | | | | |
| 20. Wound care is the cornerstone of RDEB patient treatment. The wound care plan should be individually tailored and consider psychosocial aspects and patient preferences as well as cost effectiveness. Patient and caregiver should be trained, and adherence to treatment should be regularly checked. | 73 | 27 | 0 | 0 | 0 | 5 (1) |
| 21. Appropriate analgesia must be administered before dressing changes, which should be performed in a relaxing environment with all dressing materials ready for use. | 57 | 43 | 0 | 0 | 0 | 5 (1) |
| 22. The choice of dressings should consider the wound characteristics (site, and size, exudate, critical colonization/infection), patient age, and patient/parent preference. | 60 | 37 | 3 | 0 | 0 | 5 (2) |
| 23. In the absence of infection, wound dressing should be performed 2-3 times per week, using advanced non-adherent primary dressings, such as soft silicone foams and polymeric membrane dressings for exuding wounds, and soft silicone or lipido-colloid contact layers and hydrogels for dry/slightly exuding wounds. When advanced dressings are not available, paraffin-impregnated gauzes can be used and should be changed daily. | 60 | 37 | 3 | 0 | 0 | 5 (2) |
| 24. Topical treatments comprise: i) liquid paraffin for gentle crust removal, and ii) birch triterpenes gel for wounds. | 40 | 57 | 3 | 0 | 0 | 4 (2) |
| 25. Individual digit dressing initiated in infancy should be continued to delay digit fusion. The separation should be performed by using easily modelled dressings, such as contact layers or soft silicone foams. | 60 | 40 | 0 | 0 | 0 | 5 (1) |
| 26. Treatment of critically colonized and infected wounds requires specific measures: (i) skin swab should be taken for microorganism identification and antimicrobial sensitivity determination, (ii) wound dressing should be performed daily using silicone-based foam dressings as contact layers, (iii) cleansing with mild antiseptics and application of an antiseptic cream (such as lipid-stabilized hydrogen peroxide) can reduce bacterial load, (iv) topical antibiotics/antimicrobials (e.g. fusidic acid, mupirocin) should be used for short periods to prevent resistance and sensitization, (v) silver-containing products can be alternatively employed, paying attention to limit the time of administration and treated surface due to potential systemic absorption, (vi) systemic antibiotics should be administered in the presence of multiple infected lesions and/or deep infection with surrounding tissue involvement. | 57 | 43 | 0 | 0 | 0 | 5 (1) |
| 27. Healing of infected wounds should be closely monitored by clinical evaluation of wound (exudate) and perilesional area (erythema and swelling) appearance and odor, improvement of local symptoms (pain), and then size reduction. | 60 | 40 | 0 | 0 | 0 | 5 (1) |
| **4.3 Oral and dental care** |  | | | | | |
| 28. RDEB patients experience numerous oral problems: blisters, ulcerations, inflammation, and frequently severe scarring, as well as multiple caries causing pain and impairing feeding. Oral hygiene and dental care are important aspects of RDEB management. | 77 | 20 | 3 | 0 | 0 | 5 (2) |
| 29. Preventive management should always be adopted before the teeth erupt; parents and patients should be trained to carry out all preventive measures (e.g. oral hygiene, fluoride), and visits should be regularly scheduled. | 67 | 33 | 0 | 0 | 0 | 5 (1) |
| 30. Tooth brushing technique should be individually tailored and practically demonstrated. The use of small soft or electric brushes is recommended. | 50 | 43 | 7 | 0 | 0 | 4.5 (2) |
| 31. Professional tooth cleaning should be regularly performed. | 70 | 30 | 0 | 0 | 0 | 5 (1) |
| **4.4 Gastrointestinal involvement** |  | | | | | |
| 32. Esophageal strictures are one of the most common and severe gastrointestinal complications, they can occur from early childhood. They cause chronic dysphagia and odynophagia, resulting in reduced food intake and malnutrition. Early diagnosis and appropriate management are crucial aspects of RDEB care. | 73 | 27 | 0 | 0 | 0 | 5 (1) |
| 33. In patients with suggestive clinical signs and symptoms, esophageal stricture diagnosis should be confirmed radiologically with contrast studies (esophagogram, videofluoroscopy) paying attention to the upper esophagus where strictures are more frequently located. | 60 | 40 | 0 | 0 | 0 | 5 (1) |
| 34. Non-pharmacological measures to prevent/delay progression of esophageal strictures comprise dietary modifications (soft, non-spicy food), and adequate dental and oral care. | 63 | 37 | 0 | 0 | 0 | 5 (1) |
| 35. In patients with confirmed diagnosis, esophageal dilation should be performed in a reference center, preferably by fluoroscopically-guided balloon dilation. Stricture relapses are common and can be treated by repeated dilations. | 70 | 30 | 0 | 0 | 0 | 5 (1) |
| **4.5 Nutrition** |  | | | | | |
| 36. The maintenance of an adequate nutritional status is extremely challenging in RDEB patients, and requires the constant involvement of a dietitian and a nutritionist in the follow-up of the patient. | 70 | 27 | 3 | 0 | 0 | 5 (2) |
| 37. Nutritional support generally aims to: i) improve feeding and minimize nutritional deficiencies, ii) ameliorate growth, iii) optimize bowel function, iv) improve wound healing, v) promote pubertal development and sexual maturation. | 77 | 23 | 0 | 0 | 0 | 5 (1) |
| 38. In the absence of specific data in RDEB, energy requirements may be estimated starting from those of age/height- and gender-matched unaffected children, with the addition of factors that consider the extent of skin lesions, presumed level of bacterial infection, and requirement for catchup growth. Practically, the energy requirement usually ranges from 100% to 150% of the estimated average for normal children. | 63 | 37 | 0 | 0 | 0 | 5 (1) |
| 39. Biochemical and hematological parameters to identify deficient micronutrients and vitamins that require supplementation [in particular zinc, selenium, vitamins C, 25(OH)D3, K, niacin, B6, and B12] should be regularly evaluated. | 57 | 33 | 10 | 0 | 0 | 5 (2) |
| 40. In RDEB children with insufficient growth, gastrostomy placement is recommended. | 50 | 50 | 0 | 0 | 0 | 4.5 (1) |
| 41. Constipation should be promptly and regularly treated with increased fluid and fiber intake, and administration of macrogol (polyethylene glycol), when required. | 57 | 43 | 0 | 0 | 0 | 5 (1) |
| **4.6 Anemia** |  | | | | | |
| 42. Anemia is a common complication of RDEB. The etiology is multifactorial, with iron deficiency and chronic inflammation being the primary factors. Iron deficiency is due to iron losses from chronic bleeding wounds, and poor dietary intake and absorption. Early diagnosis and management of iron deficiency and anemia are crucial to decrease anemia-related symptoms, promote wound healing, increase growth, and improve quality of life. | 80 | 20 | 0 | 0 | 0 | 5 (1) |
| 43. For generalized RDEB forms, anemia should be evaluated twice a year starting from infancy. | 57 | 43 | 0 | 0 | 0 | 5 (1) |
| 44. The gold standard for diagnosis of anemia is hemoglobin (Hb) level. Diagnosis and severity of anemia will be based on the WHO recommendations. Low ferritin level and elevated total iron-binding capacity (TIBC) can support diagnosis of iron deficiency. | 53 | 40 | 7 | 0 | 0 | 5 (2) |
| 45. Treatment includes dietary measures, iron supplementation and blood transfusion. Oral iron can be administered in mild anemia (Hb levels >10 g/dL); iron infusion is reserved for moderate to severe anemia (Hb level <10 g/dL), or in patients who do not tolerate oral iron. Blood transfusion should be performed if Hb is <8 g/dL in adults and <6 g/dL in children. | 46 | 47 | 7 | 0 | 0 | 4 (2) |
| **4.7 Ocular involvement** |  | | | | | |
| 46. Ocular manifestations affecting the conjunctiva, cornea and eyelids are common in RDEB (> 50% of patients). They comprise chronic blepharitis, eyelid blisters, recurrent painful corneal erosions leading to scarring, opacities, impaired vision and, rarely, blindness. All patients should be referred to the ophthalmologist of a reference center for a baseline examination and should be followed as frequently as deemed necessary according to the severity of ocular findings. | 70 | 30 | 0 | 0 | 0 | 5 (1) |
| 47. Secondary corneal dryness is treated with sterile ophthalmologic lubricating ointments and preservative-free artificial tears. Practical measures to reduce tear film evaporation include bedroom humidifiers. | 53 | 47 | 0 | 0 | 0 | 5 (1) |
| **4.8 Hand and foot deformities** |  | | | | | |
| 48. In RDEB patients, hands and feet are particularly prone to repeated blistering, ulceration and scarring. Hand deformities comprise thumb adduction contractures, digit pseudosyndactyly, and flexion contractures of all joints including the wrist. They are almost constant in severe RDEB, and result in major functional impairment. Thus, a continuous management is required. | 73 | 27 | 0 | 0 | 0 | 5 (1) |
| 49. Treatments are aimed at delaying deformities and contractures with medical and occupational therapy/physiotherapy; improving function with surgery; delaying recurrence with splinting and meticulous skin care. | 73 | 27 | 0 | 0 | 0 | 5 (1) |
| 50. Hand surgery results in functional improvement which is always temporary. Before any surgery is planned, the patient and family members must be fully informed on surgical procedure, complications (pain, bleeding, infections) and constant relapses, and necessity of hand physiotherapy and splinting to delay relapse after surgery. | 70 | 30 | 0 | 0 | 0 | 5 (1) |
| 51. In addition to blistering and scars, RDEB podiatric manifestations include nail dystrophy and structural abnormalities/deformities affecting foot positioning. Generally, the management tends to be supportive and aims to prevent blistering by providing information on suitable shoes, cushioning materials, and appropriate insoles or orthotics. Physiotherapy and occupational therapy are crucial to improve and maintain mobility. | 70 | 30 | 0 | 0 | 0 | 5 (1) |
| **4.9 Cutaneous squamous cell carcinoma** |  | | | | | |
| 52. All RDEB subtypes are associated with an increased risk of developing cutaneous squamous cell carcinomas (cSCCs). This epithelial skin cancer is characterized by a rapid growth and an aggressive biological behavior and represents the first cause of mortality in RDEB patients. Early cSCC diagnosis is a crucial aspect of RDEB care. | 73 | 27 | 0 | 0 | 0 | 5 (1) |
| 53. Clinical diagnosis of cSCC may be difficult as this cancer usually develops on chronic wounds. Suggestive clinical features are: (i) non-healing chronic wounds despite adequate treatment, (ii) rapid wound enlargement, (iii) deep wound with raised or rolled edges, (iv) exuberant/vegetating appearance, (v) areas of thick hyperkeratosis, and (vi) increased wound pain or a tingling sensation. | 67 | 33 | 0 | 0 | 0 | 5 (1) |
| 54. Development of cSCC may occur starting from the second decade of life in patients with severe RDEB. Thus, total body skin examination should be performed by an expert dermatologist from an EB reference center every 3–6 months starting from 9-10 years of age. Patients with a history of cSCC should be evaluated at 3-month intervals. | 60 | 40 | 0 | 0 | 0 | 5 (1) |
| 55. Diagnostic biopsies for histopathological examination should always be performed in suspicious areas. To reduce the risk of misdiagnosis, multiple biopsies should be taken. | 64 | 30 | 3 | 3 | 0 | 5 (3) |
| 56. Wide surgical excision is the first-line treatment for RDEB cSCC. The surgical approach is defined by the surgeon/plastic surgeon, in collaboration with the dermatologist and oncologist, taking into account the anatomical location and size of the lesion, RDEB skin fragility, as well as patient preference. | 70 | 27 | 3 | 0 | 0 | 5 (2) |
| 57. Alternative therapeutic options including radiotherapy, chemotherapy, electrochemotherapy or targeted therapy with EGFR inhibitors should be considered when surgical excision is not feasible, and in locally advanced or metastatic disease. At present, immunotherapy with programmed cell death protein 1 inhibitors is approved for metastatic and locally advanced cSCC. It has been employed in a limited number of RDEB patients providing some clinical benefit. | 60 | 40 | 0 | 0 | 0 | 5 (1) |
| **4.10 Delayed puberty and osteoporosis** |  | | | | | |
| 58. Pubertal delay is highly prevalent in RDEB, involving one third to a half of patients, and being mainly related to malnutrition and inflammatory status. From 9-10 years of age, RDEB patients should be regularly evaluated by the endocrinologist at the reference center to early detect delay of puberty. | 70 | 27 | 3 | 0 | 0 | 5 (2) |
| 59. Low bone mineral density is reported in about 30% of RDEB pediatric patients. Osteoporosis has been detected in 75% of severe RDEB adults. Evaluation of bone mineral density should be considered starting from adolescence in patients with severe RDEB. | 56 | 37 | 7 | 0 | 0 | 5 (2) |
| 60. Preventive measures should be taken from early childhood to favor bone growth and prevent osteopenia/osteoporosis. Adequate nutrition, calcium and vitamin D supplementation as well as physiotherapy are recommended. | 80 | 20 | 0 | 0 | 0 | 5 (1) |
| **4.11 Renal involvement** |  | | | | | |
| 61. RDEB patients may develop renal involvement, which can progress to renal failure, leading to a significant increase of morbidity and even mortality. Evaluation of renal function should be performed every 6-12 months as part of RDEB patient follow-up. We suggest to evaluate plasma creatinine (and cystatin C in case of malnutrition), urinalysis and blood pressure. In case of abnormal findings, nephrologist should be promptly involved. | 73 | 27 | 0 | 0 | 0 | 5 (1) |
| 62. Hemodialysis and peritoneal dialysis are both reported in RDEB patients. Dermatologists and nephrologists should evaluate case-by-case the most suitable dialysis modality, when needed. | 57 | 43 | 0 | 0 | 0 | 5 (1) |
| **4.12 Sexuality, pregnancy and delivery** |  | | | | | |
| 63. RDEB manifestations and complications have a high impact on sexual life. Multidisciplinary team members should consider and address psychosocial and medical issues related to sexuality and pubertal/sexual development. | 67 | 33 | 0 | 0 | 0 | 5 (1) |
| 64. Pregnancy has been described in women with RDEB. However, nutritional compromise, low body mass index, and delayed puberty may affect fertility in these patients. Pre-pregnancy diet and nutrition optimization may improve maternal and perinatal outcomes. | 53 | 37 | 10 | 0 | 0 | 5 (2) |
| 65. The multidisciplinary team members who should be early involved in management of pregnancy and delivery are obstetrician, midwife, anesthetist, clinical nurse specialist, dermatologist, nutritionist, and psychologist. | 53 | 47 | 0 | 0 | 0 | 5 (1) |
| 66. Although RDEB is not an absolute contraindication to vaginal birth, an individualized birth plan should be discussed among the multidisciplinary team members. Instrumental delivery, including vacuum suction or forceps-assisted delivery should be avoided, whenever possible. | 60 | 37 | 3 | 0 | 0 | 5 (2) |
| **5. TRANSVERSAL AGE-INDEPENDENT ISSUES** |  | | | | | |
| **5.1 Pain and itch management** |  | | | | | |
| 67. Pain is one of the most common and disabling symptoms in RDEB patients starting from the first days of life. Pain is primarily related to mucocutaneous wounds, but also to different disease complications (e.g. GERD, constipation, joint contractures). Patients suffer from acute and chronic pain that exacerbates during each procedure (e.g. bathing, wound dressing), and need adequate treatment. | 80 | 20 | 0 | 0 | 0 | 5 (1) |
| 68. RDEB patient pain is nociceptive, neuropathic, and psychogenic. Pain memory in children leads to increased future pain intensity fear and distress. Thus, pain should be promptly assessed and treated by the pain therapist. | 77 | 23 | 0 | 0 | 0 | 5 (1) |
| 69. For mild pain (pain-NRS or FLACC <4/10), non-opioid analgesics (e.g. acetaminophen, ibuprofen) can be used. Moderate or severe pain requires an opioid analgesic (e.g. nefopam, tramadol, morphine, oxycodone, methadone). Tricyclic antidepressants (e.g. amitriptyline) or anti-epileptics (e.g. gabapentin) can be associated for chronic pain. | 60 | 40 | 0 | 0 | 0 | 5 (1) |
| 70. Non-pharmacological and psychological therapies, including cognitive behavioural therapy, hypnosis, biofeedback and relaxation techniques, may all contribute to reduce pain intensity and related distress, and to improve pain coping and quality of life. | 47 | 53 | 0 | 0 | 0 | 4 (1) |
| 71. Additional analgesia should be regularly administered before dressing changes which should be carried out in a relaxing context. | 63 | 37 | 0 | 0 | 0 | 5 (1) |
| 72. Itch (pruritus), usually chronic, is also a common symptom, with a major impact on quality of life. It occurs more frequently at wound sites but may also be generalized, and should be adequately managed. | 63 | 37 | 0 | 0 | 0 | 5 (1) |
| 73. Topical therapies comprise bathing in tepid water with syndet/oil cleanser, skin hydration with emollients, and short courses of corticosteroids. Widely used systemic treatments are sedating and non-sedating antihistamines, tricyclic antidepressants (amitriptyline, doxepin) and anticonvulsants (gabapentin, pregabalin). | 53 | 40 | 7 | 0 | 0 | 5 (2) |
| **5.2 Patient care in the operating theatre** |  | | | | | |
| 74. In RDEB patients, surgery in sedation/general anesthesia should be limited to strictly necessary procedures following discussion among multidisciplinary team members. | 73 | 27 | 0 | 0 | 0 | 5 (1) |
| 75. The anesthetic team should be adequately trained to avoid rubbing or stroking patient skin and mucosae prior to and during surgery (e.g. padding of frictional/pressure areas, avoidance of all adhesive materials, patient moving using a blanket, surgical site disinfection by solution pouring or gentle dabbing, lubrication of all airway equipment). In parallel, the operating theatre should be adequately equipped (e.g. antidecubitus mattress, non-adherent silicon-based dressings and tapes, clip-type pulse oximetry). | 73 | 27 | 0 | 0 | 0 | 5 (1) |
| 76. RDEB patient airway management can be particularly challenging due to microstomia and oro-pharyngeal scarring: in these cases, the Ear Nose and Throat specialist should be included in the anesthetic team. | 60 | 33 | 7 | 0 | 0 | 5 (2) |
| **5.3 Physiotherapy and occupational therapy** |  | | | |  |  |
| 77. Due to fibrosis, scarring, joint contractures and pain, RDEB patients can manifest reduced functional mobility and limited motor skills, with a major impact on daily life. The physiotherapist should be early involved. | 63 | 37 | 0 | 0 | 0 | 5 (1) |
| 78. The physiotherapist should assess functional ability and aim to optimize: i) developmental motor milestone attainment, ii) safe and functional mobility, iii) ambulation endurance, iv) ability to safely bear weight, and v) interaction with environment. | 67 | 33 | 0 | 0 | 0 | 5 (1) |
| 79. In addition to the physiotherapist, an occupational therapist may be involved in RDEB patient care with the aim to optimize independence and preserve quality of life. | 67 | 33 | 0 | 0 | 0 | 5 (1) |
| 80. Occupational therapy interventions may help improving patients' abilities in daily activities, hand function, fine motor development and retention, as well as oral feeding skills. | 70 | 30 | 0 | 0 | 0 | 5 (1) |
| **5.4 Therapeutic patient education** |  | | | | | |
| 81. Therapeutic patient education (TPE) is a continuous process of patient-centered medical care, enabling patients affected by chronic diseases, and their families, to better manage their illness and, overall, improving adherence to treatment. The process is dynamic: it should consider patient age, modification of clinical features and complications in disease course, and consequent psychological impact. | 67 | 30 | 3 | 0 | 0 | 5 (2) |
| 82. Before infant discharge, parents/caregivers should be clearly informed about RDEB and trained to take care of all disease aspects from wound management to nutrition. | 80 | 20 | 0 | 0 | 0 | 5 (1) |
| 83. During subsequent follow-ups, TPE should be periodically checked and updated with the involvement of the dermatologist and specialized nurse for wound and skin care, which represents the most complex and time-consuming aspect of disease management, as well as of the pediatrician and nutritionist/dietitian for nutrition. The support of a psychologist in delivering TPE is desirable. Other specialists will be involved overtime depending on disease manifestations and complications. | 64 | 33 | 3 | 0 | 0 | 5 (2) |
| 84. Age-appropriated TPE should be delivered also to the child starting from about 5 years of age. During adolescence, compliance to treatment usually decreases, while complications increase (including the risk of developing cSCC), making TPE more challenging but also more essential. | 67 | 33 | 0 | 0 | 0 | 5 (1) |
| **5.5 Psychosocial support** |  | | | |  |  |
| 85. RDEB has a profound impact on all domains of patient and family life, including social interactions, education, employment and leisure. Thus, lifelong psychosocial support should be guaranteed to patients, in order to improve their quality of life (QoL) and well-being, and to help them coping with the disease. | 73 | 27 | 0 | 0 | 0 | 5 (1) |
| 86. Psychosocial care for family and caregivers is also recommended to improve their QoL and well-being and to prevent family breakdown. | 80 | 20 | 0 | 0 | 0 | 5 (1) |
